# Is there any role of thrombin activatable fibrinolysis inhibitor in the development of a hypercoagulable state in gastric cancer

**DOI:** 10.1186/1477-7819-10-180

**Published:** 2012-08-31

**Authors:** Mehmet Eser, Metin Kement, Salim Balin, Cihan Coskun, Umut Kefeli, Mahmut Gumus, Yunus Emre Altuntas, Necmi Kurt, Alparslan Mayadagli

**Affiliations:** 1 Department of General Surgery, Kartal Education and Research Hospital, Istanbul, Turkey; 2 Department of Biochemistry, Kartal Education and Research Hospital, Istanbul, Turkey; 3 Department of Medical Oncology, Kartal Education and Research Hospital, Istanbul, Turkey; 4 Department of Radiation Oncology, Kartal Education and Research Hospital, Istanbul, Turkey

**Keywords:** Gastric cancer, TAFI, Hypercoagulation

## Abstract

**Background:**

The purpose of this study was to investigate plasma levels of thrombin activatable fibrinolysis inhibitor (TAFI) and TAFI’s relationship with coagulation markers (prothrombin fragment 1 + 2) in gastric cancer patients.

**Methods:**

Thirty-three patients with gastric adenocarcinoma and 29 healthy control subjects were prospectively enrolled in the study. Patients who had a history of secondary malignancy, thrombosis related disease, oral contraceptive use, diabetes mellitus, chronic renal failure or similar chronic metabolic disease were excluded from the study. A fasting blood sample was drawn from patients to determine the plasma levels of TAFI and Prothrombin Fragment 1 + 2 (F 1 + 2). In addition, data on patient age, sex, body mass index (BMI) and stage of disease were recorded. The same parameters, except stage of disease, were also recorded for the control group. Subsequently, we assessed the difference in the levels of TAFI and F 1 + 2 between the patient and control groups. Moreover, we investigated the relation of TAFI and F 1 + 2 levels with age, sex, BMI and stage of disease in the gastric cancer group.

**Results:**

There were no statistical differences in any demographic variables (age, gender and BMI) between the groups (Table 1). The mean plasma TAFI levels of the gastric cancer group (69.4 ± 33.1) and control group (73.3 ± 27.5) were statistically similar (*P* = 0.62). The mean plasma F 1 + 2 level in the gastric cancer group was significantly higher than for those in the control group (549.7 ± 325.3 vs 151.9 ± 67.1, respectively; *P* < 0.001). In the gastric cancer group, none of the demographic variables (age, gender and BMI) were correlated with either TAFI or F 1 + 2 levels. Also, no significant associations were found between the stage of the cancer and either TAFI or F 1 + 2 levels.

**Conclusion:**

In our study, TAFI levels of gastric cancer patients were similar to healthy subjects. The results of our study suggest that TAFI does not play a role in pathogenesis of the hypercoagulable state in gastric cancer patients.

## Background

The association between cancer and venous thromboembolism (VTE) is well-established. The overall risk of thrombosis in cancer patients is seven-fold that of non-cancer patients and up to 20% of cancer patients develop VTE. Venous thromboembolism (VTE), which includes both deep vein thrombosis (DVT) and pulmonary embolism (PE), is the second leading cause of death in hospitalized and ambulatory cancer patients [1-4]. The probability of thrombosis occurring in a cancer patient is dependent on several factors, including the type of cancer, the clinical stage, accompanying medical problems, performance status and the treatment modalities employed. Tumor cells can stimulate blood coagulation through various mechanisms, including production of pro-coagulant, fibrinolytic and pro-aggregating activities; release of pro-inflammatory and pro-angiogenic cytokines; and direct interaction with blood and host vascular cells (for example, platelets, leukocytes and endothelial cells) and through adhesion molecules.

Thrombin Activatable Fibrinolysis Inhibitor (TAFI) is a single chain glycoprotein zymogen (Mr = 60,000) synthesized in the liver and circulating at a plasma concentration of 50 nM. TAFI is proposed to play a key role in the interactions among procoagulant, anticoagulant and fibrinolytic systems [5-9]. Effective fibrinolysis results from the formation of a ternary complex among tPA, plasminogen and C-terminal lysine residues on fibrin. Plasminogen bound to fibrin is more effectively converted to plasmin, thereby localizing the lytic activity to the area of the clot. Plasmin degradation of fibrin generates additional C-terminal lysine residues thereby amplifying the system locally. The ability of TAFI to bind specifically to plasminogen and to cleave C-terminal lysines on fibrin (and cell surfaces) results in down-regulation of fibrinolysis by reducing the number of plasminogen and tPA binding sites on fibrin. The activation of TAFI by the thrombin/thrombomodulin complex couples both the phenomenon of coagulation induced inhibition of fibrinolysis and the profibrinolytic effect of activated protein C. Increased plasma levels of TAFI were reported to be a contributing factor of thrombotic disorders in some kinds of cancers, such as breast and lung cancers.

The aim of the present study was to investigate plasma levels of TAFI and TAFI’s relationship with coagulation markers (prothrombin fragment 1 + 2) in gastric cancer patients.

## Methods

### Study design

Thirty-three patients, who presented with the diagnosis of gastric adenocarcinoma between January and December 2011 in the General Surgery and Medical Oncology Clinics of our institution, were prospectively enrolled in the study. Patients with a history of secondary malignancy, thrombosis related disease, oral contraceptive use, diabetes mellitus, chronic renal failure or similar chronic metabolic disease were excluded from the study. The control group consisted of 29 healthy subjects with similar demographic characteristics to the gastric cancer group. All subjects gave informed consent to participate in the study and the study protocol was approved by the institutional ethical committee.

A fasting blood sample was drawn from patients to determine the plasma levels of TAFI and prothrombin fragment 1 + 2 (F 1 + 2). In addition, data on patient age, sex, body mass index (BMI) and stage of disease were recorded. The same parameters, except stage of disease, were also recorded for the control group. Subsequently, we assessed the difference in the levels of TAFI and F 1 + 2 between the patient and control groups. Moreover, we investigated the relation of TAFI and F 1 + 2 levels with age, sex, BMI and stage of disease in the patient groups.

### Measurement methods

Blood samples were collected by atraumatic venipuncture without pressure into plastic tubes containing 0.129 M/0,109 M trisodium citrate anticoagulant (using a ratio of 9:1 volumes).

Thrombin-activatable fibrinolysis inhibitor functional activity was assayed using the Actichrome® TAFI Activity kit **(**American diagnostica Inc., CT, USA). The TAFI level is determined by first incubating the plasma with a TAFI Activation Reagent, a specially formulated thrombin/thrombomodulin complex that converts the TAFI to its activated form, TAFIa. An Activation Stop Reagent is then added to halt the activation step. Next, the TAFI Developer containing the substrate is added to the plasma sample and an enzymatic reaction begins. The reaction is stopped by the addition of sulfuric acid and the absorbance of the solution is read in a spectrophotometer at 490 nm. Plasma that was not activated (not incubated with the TAFI Activation Reagent) is assayed in parallel as a control. The difference in absorbance between the activated and non-activated plasma, ΔA490, is calculated and represents the amount of TAFI activity in the sample. The TAFI concentration is determined by directly interpolating the ΔA490 with the absorbances generated by a TAFIa standard curve.

F 1 + 2 was measured with a sandwich enzyme–linked immunosorbent assay method (Enzygnost F 1 + 2 micro). The sandwich assay makes use of two polyclonal antibodies against prothrombin and F 1 + 2. The catching antibodies, which are immobilized on microtiter plates, were raised in rabbits against a 14 amino acid negatively charged synthetic peptide corresponding to the C-terminal part of F 1 + 2. They do not cross-react with intact prothrombin. The other antibody (polyclonal, rabbit) reacts with prothrombin and F 1 + 2. It has been coupled to peroxidase. The enzyme activity is determined with the substrate o-phenylenediamine in the presence of hydrogen peroxide. The test kit Enzygnost F 1 + 2 micro (Dade Behring, Marburg, Germany), contains all the necessary reagents to perform the assay. The standard curve ranges from 0.04 to 10 nmol/L. The F 1 + 2 concentration is determined by directly interpolating the ΔA492 (490 to 500 nm) with the absorbances generated by a F 1 + 2 standard curve.

### Statistic analysis

Statistical analysis was performed using SPSS for Windows 17 (SPSS Inc., IL,USA). Data are presented as mean ± standard deviation and percentage. The groups were compared using the Chi-square test and the Mann–Whitney *U* test for categorical or continuous variables, respectively. *P*-values less than 0.05 were considered statistically significant.

## Results

There were no statistical differences in any demographic variables (age, gender and BMI) between the groups (Table 1). The mean plasma TAFI levels of the gastric cancer group (69.4 ± 33.1 ng/ml) and the control group (73.3 ±27.5 ng/ml) were statistically similar (*P* = 0.62) (Table 2). The mean plasma F 1 + 2 level in the gastric cancer group was significantly higher than those in the control group (549.7 ± 325.3 pmol/l vs 151.9 ± 67.1 pmol/l, respectively; *P* < 0.001) (Table 2). In the gastric cancer group, none of the demographic variables (age, gender and BMI) were correlated with either TAFI or F 1 + 2 levels (Table 3). Also, no significant associations were found between the stage of the cancer and either TAFI or F 1 + 2 levels (Table 3).

**Table 1:** Demographic characteristics of the groups

**Variables**	**Groups**		**P-value**
	**Gastric cancer**	**Control**	
**Gender (F/M)**	14/19	14/15	0.644
**Age ± SD (Years)**	61.1 ± 11.5	64.1 ± 6.8	0.235
**BMI ± SD (kg/m**^**2**^**)**	25.6 ± 2.8	26.4 ± 2.1	0.239

**Table 2:** Plasma levels of TAFI and F 1 + 2 in the groups

**Variables**	**Groups**		**P-value**
	**Gastric cancer**	**Control**	
**TAFI Level ± SD (ng/ml)**	69.4 ± 33.1	73.3 ±27.5	0.41
**F 1 + 2 Level (pmol/l)**	549.7 ± 325.3	151.9 ± 67.1	<0.001*

**Table 3:** TAFI and F 1 + 2 levels by gender, age, BMI and stage in the gastric cancer group

		**TAFI Level**	***P*-value**	**F 1 + 2 Level**	***P*-value**
**Gender**	**Female**	63.6 ± 22.7	0.32	483.7 ± 230.8	0.28
	**Male**	73.6 ± 39.1		598.3 ±379.2	
**Age**	**>60 (n = 21)**	67.7 ± 32.1	0.52	614 ± 337.8	0.11
	**≤60 (n = 12)**	73.5 ± 37.1		437.2 ± 280.5	
**BMI**	**>25 (n = 15)**	65.7 ± 31.8	0.34	583.7 ± 328.7	0.47
	**≤25 (n = 18)**	74.8 ± 34.8		521.3 ± 329.1	
**Stages**	**Stage 1a (n = 1)**	93.3	0.92	484	0.81
	**Stage 1b (n = 0)**	-		-	
	**Stage 2 (n = 5)**	68.2 ± 21.3		416.8 ± 80.9	
	**Stage 3a (n = 14)**	69 ± 23.		609.5 ± 354.7	
	**Stage 3b (n = 4)**	71.8 ± 34		575 ± 498.7	
	**Stage 4 (n = 9)**	66.9 ± 51.9		526.6 ± 321	

## Discussion and conclusion

Cancer-related thrombosis was first recognized by Bouillard in 1823 and then described by Trousseau in 1844. Many studies have since provided significant evidence for a relationship between thrombosis and cancer. The relationship between the hemostatic system and cancer was shown by a study conducted by Miller *et al.*[10], which investigated hemostatic status every year for four years in a population of about 3,000 middle-aged men without cancer. Among patients with activation of the hemostatic system (defined as persistent elevation of F 1 + 2 levels and fibrinopeptide A), total mortality was considerably higher in participants with constant activation (17.1/1,000 person-years) than in patients without activation (9.7/1,000 person-years; *P* = 0.015). This difference was attributable to an increased incidence of death from cancers (11.3/1,000 vs. 5.1/1,000 person-years), mainly due to a three-fold higher mortality from cancers of the digestive tract (6.3/1,000 vs. 1.9/1,000 person-years).

Thrombin activatable fibrinolysis inhibitor (TAFI), also known as plasma procarboxypeptidase B or U, is a 60 kD glycoprotein [5-9]. The main source of TAFI production is liver cells under physiological conditions, and it may act as an acute phase reactant during inflammation, as demonstrated in mice [11,12]. In addition to liver cells, adipocytes, endothelial and epithelial cells may also produce and secrete TAFI *in vitro*[13,14]. TAFI returns the active carboxypeptidase B or U form (TAFIa, 35.8 kDa), and modulates fibrinolysis *in vivo* by cleaving the C-terminal lysine residues from partially degraded fibrin [6,9]. The reduction of the C-terminal lysine residues could thus inhibit the augmentation of plasminogen activation by tissue plasminogen activator (t-PA). Moreover, plasmin could also activate TAFI to TAFIa and inactivate TAFI to a 44.3-kDa fragment, depending on the cleavage site [10]. Therefore, TAFI can be influenced by both coagulation and fibrinolysis, especially when hemostasis is disrupted. Various pathological conditions, including hemophilia, coronary heart disease (CHD), disseminated intravascular coagulation (DIC), deep venous thrombosis (DVT) and malignancies result in different changes in TAFI levels [15-19]. Elevated TAFI levels were observed in CHD and DVT, which are thought to be caused by increased levels of coagulation factors and consequently increased fibrin clot formation [19,20]. DIC is also characterized by increased coagulation throughout the body. Reduction of TAFI levels was reported in DIC as well as in sepsis, in which a significant depletion of TAFI was observed in the presence of pathogens in plasma [16]. These results suggest that the consumption of TAFI is an important contributing factor in the pathogenesis of DIC and sepsis.

Hataji *et al.* reported that the plasma level of TAFI was significantly increased in lung cancer patients compared to healthy subjects. In their study, the concentration of TAFI was particularly found higher in patients with small cell carcinoma compared to those with adenocarcinoma or squamous cell carcinoma, and in cancer patients that responded to chemotherapy compared to non-responders [21]. Similar to Hataji *et al.*, Koldas *et al.* found increased circulating levels of TAFI in patients with non-small cell lung cancer. However, in their study, there were no statistically significant relationships between TAFI levels and patient age, sex, BMI, histopathology or stage of disease. Balcik *et al.* reported significantly elevated TAFI levels in patients with multiple myeloma. Futhermore, they demonstrated that higher TAFI levels were associated with a more advanced disease stage [22]. Likewise, Kaftan *et al.* reported that the plasma level of TAFI was significantly increased in breast cancer patients compared to healthy subjects [23].

The mechanism of increased circulating levels of TAFI in cancer patients is not clear. Inflammatory cytokines induced by malignant cells may stimulate the production and secretion of TAFI from liver or vascular endothelial cells and thus increase its systemic circulation. Besides, Hataji *et al.* reported that lung cancer cell lines, particularly small cell lung cancer cell lines, can express the mRNA and protein of TAFI, suggesting that malignant cells may also be a direct source of TAFI in cancer patients [21]. In addition to causing systemic activation of the blood coagulation system, secretion of TAFI from cancer cells may increase intra-tumoral fibrin deposition and thus promote the growth and dissemination of tumor cells [24]_._

The prothrombin fragment 1–2 (F 1 + 2) is released when activated factor X cleaves prothrombin to thrombin, and it reflects the *in vivo* thrombin generation. F 1 + 2 were demonstrated as independent and predictive of the occurrence of VTE in cancer patients [25].

To our knowledge, the present study is one of the first to investigate the relationship between gastric cancer and TAFI levels. We found significantly higher F 1 + 2 levels in gastric cancer patients compared to healthy subjects, suggesting that the risk of thrombosis in gastric cancer patients was markedly increased. However, in our study, the TAFI levels of gastric cancer patients were similar to healthy subjects. The results of our study suggest that TAFI does not play a role in pathogenesis of the hypercoagulable state in gastric cancer patients. Contrary to this, in a very recent study by Fidan *et al.*, TAFI levels were found to be higher in patients with gastric cancer than in healthy control subjects [26].

As conclusion, the results of our study demonstrated that the risk of thrombosis was markedly increased in gastric cancer patients. However, in these patients, any relationship between increased risk of thrombosis and TAFI levels could not be found. Even though much has been elucidated about the pathogenesis of cancer-related thrombosis, we are indeed beginning to comprehend the relationship between cancer cells and their related microenvironment, and in our opinion, such research is likely to increase our knowledge of cancer-related thrombosis mechanisms.

## Abbreviations

BMI: Body mass index; CHD: Coronary heart disease; DVT: Deep vein thrombosis; F 1 + 2: Prothrombin Fragment 1 + 2; PE: Pulmonary embolism; TAFI: Thrombin activatable fibrinolysis inhibitor; VTE: Deep vein thrombosis.

## Competing interests

The authors declare that they have no conflicts of interest concerning this article.

## Authors’ contributions

EM, KM and GM are responsible for the study conception and design. EM, BS, CC, KU and AYE acquired the data. MA and KN performed critical revisions of the manuscript. All authors read and approved the final manuscript.
